# Shear wave elastography based analysis of changes in fascial and muscle stiffness in patients with chronic non-specific low back pain

**DOI:** 10.3389/fbioe.2024.1476396

**Published:** 2024-11-15

**Authors:** Kun Liu, Tong Zhao, Yang Zhang, Lili Chen, Haoran Zhang, Xiqiang Xu, Zenong Yuan, Qingyu Zhang, Jun Dong

**Affiliations:** ^1^ Rehabilitation and Physical Therapy Department, Shandong University of Traditional Chinese Medicine Affiliated Hospital, Jinan, China; ^2^ College of Sports and Health, Shandong Sport University, Jinan, Shandong, China; ^3^ Shandong Academy of Occupational Health and Occupational Medicine, Shandong First Medical University and Shandong Academy of Medical Sciences, Jinan, Shandong, China; ^4^ Department of Orthopedics, Shandong Provincial Hospital Affiliated to Shandong First Medical University, Jinan, Shandong, China

**Keywords:** shear wave elastography, low back pain, thoracolumbar fascia, erector spinae, multifidus

## Abstract

**Background:**

The quantitative assessment of individual muscle and fascial stiffness in patients with low back pain remains a challenge. This study aimed to compare the stiffness of the thoracolumbar fascia (TLF), erector spinae (ES), and multifidus (MF) in patients with and without chronic non-specific low back pain (CNLBP) using shear wave elastography (SWE). It also sought to explore the relationship between muscle and fascial stiffness and the levels of pain and dysfunction in patients with CNLBP.

**Methods:**

In this cross-sectional study, 30 patients with CNLBP (age 27.40 ± 4.57 years, 19 males, 11 females, BMI 22.96 ± 2.55 kg/m^2^) and 32 healthy controls (age 27.94 ± 4.94 years, 15 males, 17 females, BMI 22.52 ± 2.26 kg/m^2^) were enrolled. Stiffness of the TLF, ES, and MF was measured using SWE, and Young’s modulus values were recorded. The numeric rating scale (NRS) for quantifying pain intensity and the Oswestry Disability Index (ODI) scores were recorded for the case group to examine their correlations with the resilience index.

**Results:**

The CNLBP group exhibited significantly higher shear modulus values at the L_4-5_ bilateral TLF (left: *p* = 0.014, *d* = 0.64; right: *p* = 0.002, *d* = 0.86), ES (left: *p* = 0.013, *d* = 0.66; right: *p* = 0.027, *d* = 0.58), and MF (left: *p* = 0.009, *d* = 0.69; right: *p* = 0.002, *d* = 0.85) compared to the control group. Comparable findings were observed for the right ES (*p* = 0.026, *d* = 0.59) and left MF (*p* = 0.020, *η* ^*2*^ = 0.09) at L_1-2_. Strong correlations were observed between the shear modulus of the bilateral TLF (left: *r* = 0.57, *p* = 0.001; right: *r* = 0.65, *p* < 0.001) at L_4-5_ and the NRS scores. Moderate correlations were noted between the shear modulus of the ES (left: *r* = 0.42, *p* = 0.022; right: *r* = 0.48, *p* = 0.007) and MF (left: *r* = 0.50, *p* = 0.005; right: *r* = 0.42, *p* = 0.023) at L_4-5_ and the NRS scores. Additionally, the shear modulus of the MF (*r* = 0.50, *p* = 0.005) on the left side of L_1-2_ showed similar correlations. Strong correlations were observed between the shear modulus of the bilateral TLF (left: *r* = 0.60, *p* < 0.001; right: *r* = 0.58, *p* < 0.001) at L_4-5_ and the ODI scores. Moderate correlations were observed between the shear modulus of the right TLF (*r* = 0.43, *p* = 0.017), ES (*r* = 0.38, *p* = 0.037), and MF (*r* = 0.44, *p* = 0.015) at L_1-2_, as well as the bilateral MF (left: *r* = 0.46, *p* = 0.011; right: *r* = 0.45, *p* = 0.012) at L_4-5_, and the ODI scores. No significant correlations were found at other measurement sites.

**Conclusion:**

In patients with CNLBP, the stiffness of the lumbar fascia and muscles is generally higher than in individuals without LBP. However, this increase is not uniform across all lumbar regions, with the most significant changes observed in the L_4-5_ segments. In addition, higher stiffness may be associated with pain and dysfunction, primarily manifested in the TLF.

## 1 Introduction

Low back pain is a leading cause of disability and work absence, significantly impacting both individuals and society economically (Manchikanti et al., 2014). Epidemiological surveys indicate that 70%–80% of adults have experienced low back pain, establishing it as a global public health issue (Chen et al., 2022). The majority of these patients (approximately 84%) suffer from non-specific low back pain (NLBP), characterized by pain not attributable to a specific pathological cause. When this pain persists for more than 3 months, it is termed chronic non-specific low back pain (CNLBP) (Chou et al., 2007; Maher et al., 2017). Although some patients exhibit conditions like disc degeneration, lumbar disc herniation, and spinal stenosis, these conditions often do not correlate strongly with symptoms and can be present in asymptomatic individuals. Research suggests that chronic strain on the muscles and fascia of the lower back might play a crucial role in the development of CNLBP (Ouyang et al., 2013; Lemes et al., 2021). Studies have identified key pathological changes in the lumbar muscles of CNLBP patients, such as muscle fiber atrophy, degeneration, and increased fat content (Goubert et al., 2016; Seyedhoseinpoor et al., 2022). Physical examinations often reveal stiff muscles, painful nodules, and cord-like changes. However, there is a lack of objective, quantitative evaluation criteria for chronic muscle and fascia strain. Conventional imaging techniques do not reveal the mechanical properties of muscles and fascia in CNLBP, such as elasticity or tension. Moreover, the impact of muscle fiber structure changes on muscle biomechanical properties and function remains unclear (Noonan and Brown, 2021). Therefore, studying the mechanical properties of muscles and fascia, including elasticity or stiffness, could enhance our understanding of muscle fiber alterations and their relationship to both normal and impaired muscle function.

The erector spinae (ES) and multifidus (MF) muscles are crucial paraspinal muscles and core stabilizers of the lumbar spine. Current studies indicate that patients with chronic low back pain often exhibit degenerative changes in these muscles, such as atrophy and fatty infiltration (Goubert et al., 2017). Additionally, delayed pre-activation and impaired coordination control of these muscles contribute to decreased spinal stability (Zhang et al., 2021; Cheng et al., 2023). However, the physiological mechanisms underlying these phenomena, particularly the link between MF degeneration and spinal disorders, remain insufficiently explored. The thoracolumbar fascia (TLF) has also been identified as a potential source of CNLBP (Willard et al., 2012). The TLF, along with muscles and the spine, forms an extensive muscle-fascia complex system that stabilizes the thoracolumbosacral spine region and facilitates tension transmission between structures (Bojairami and Driscoll, 2022). Histological studies have shown the presence of injury-free nerve endings in the TLF and suggest that long-term muscle fatigue due to poor posture and weight-bearing can lead to microinjuries and inflammation (Sinhorim et al., 2021; Kondrup et al., 2022). These microinjuries and associated inflammation in the TLF might contribute to pain and appear to cause morphological changes in patients with chronic low back pain (Hoheisel and Mense, 2015). However, the exact relationship between these changes and the etiology of pain remains unclear.

Since Ophir et al. (Ophir et al., 1991) first used strain elastography (SE) to measure muscle stiffness *in vitro* in 1991, ultrasound elastography has enabled the study of the mechanical properties of individual muscles. However, traditional SE relies on applying pressure to the tissue with the ultrasound probe, causing tissue deformation and providing relative stiffness measurements. This quasi-static technique is heavily dependent on the examiner’s applied pressure, which can affect the accuracy of the results. Shear wave elastography (SWE), an emerging technology, overcomes this limitation by quantifying tissue stiffness through the measurement of shear wave propagation speed induced by ultrasound, without relying on manual pressure from the examiner (Taljanovic et al., 2017). SWE is non-invasive, provides real-time, dynamic quantitative data on soft tissue biomechanics, and generates qualitative elastography maps that visually depict tissue stiffness changes (Klauser et al., 2014). Additionally, SWE can differentiate between different tissue layers, making it particularly useful in complex structures such as the fascia and muscles of the lower back, where it allows for precise targeting and measurement of deep muscle shear modulus. Recent studies have demonstrated that SWE has high reliability and validity in assessing the elasticity of lumbar muscles and fascia (Moreau et al., 2016; Koppenhaver et al., 2018; Chen et al., 2020).

Pirri et al. (2023) explored the morphological changes in the TLF using ultrasound imaging, revealing that, compared to individuals without LBP, patients with CNLBP exhibited significantly reduced anisotropy levels and increased thickness in the TLF. These structural changes, which they termed “frozen back,” were hypothesized to be linked to a decrease in the sliding ability between fascial layers. Immunohistochemical examination of TLF samples from two patients with chronic low back pain by Willard et al. (Willard et al., 2012) demonstrated that the density of myofibroblasts in these patients was comparable to that observed in patients with frozen shoulder, and they described this condition as “frozen lumbar.” Ranger et al. (Ranger et al., 2016) further investigated the relationship between TLF structure and low back pain, showing that shortened TLF length was closely associated with more severe low back pain. Additionally, changes in the morphology and function of the paraspinal muscles, such as muscle fiber atrophy, degeneration, and fatty infiltration, have been identified as key contributing factors in CNLBP (Goubert et al., 2016; Goubert et al., 2017). Since muscles possess viscoelastic properties, when muscle tissue is replaced by fat or other scar connective tissues, their viscoelasticity is inevitably affected. As these physical properties change, the ability of the tissue to transmit mechanical waves is also impacted (Amir et al., 2022). Therefore, this study aims to assess changes in the stiffness of lumbar fascia and muscles in CNLBP patients using SWE and explore the relationship between these changes and pain severity as well as functional disability. We hypothesize that lumbar fascia and muscle stiffness is higher in CNLBP patients compared to individuals without LBP, and that this increased stiffness is significantly correlated with pain intensity and functional disability.

## 2 Materials and methods

### 2.1 Participants

The sample size was determined using G*Power 3.1 software, based on data from a pilot study and relevant literature (Koppenhaver et al., 2020). The pilot study, which included 14 participants (7 CNLBP patients and seven healthy controls), identified an effect size of *d* = 0.86 for key variables, including the stiffness of the thoracolumbar fascia, erector spinae, and multifidus muscles. The effect size was further validated by calculating values from comparable studies. Additionally, the pilot study revealed a minimum *r* ^*2*^ = 0.25 between the primary outcome variables (fascia or muscle stiffness and pain scores). Based on these results, a minimum of 21 participants per group is required to achieve an alpha level of 0.05 and a statistical power of 0.80.

From October 2022 to November 2023, patients with chronic non-specific low back pain (CNLBP) were prospectively recruited from the outpatient department of spine surgery in a tertiary hospital. The inclusion criteria were as follows: (1) diagnosis of CNLBP; (2) aged 18–40 years; (3) duration of low back pain for more than 3 months; (4) individuals without difficulty in comprehension during communication with researchers; (5) signed informed consent. The diagnosis of CNLBP was confirmed by an experienced spine surgeon. The diagnostic criteria for CNLBP were formulated with reference to relevant guidelines and included (Chou et al., 2007): (1) persistent or paroxysmal low back pain lasting more than 12 weeks; (2) the pain site located below the 12th rib cartilage and above the gluteal stripe; (3) absence of specific pathological factors; (4) no amplification and prolongation of pain due to psychosocial factors. Participants were excluded from the study if they met any of the following criteria: (1) pain caused by specific factors, such as lumbar disc herniation compressing nerve roots, lumbar spondylolisthesis, fracture, tumor, inflammatory disease, osteoporosis, etc.,; (2) presence of serious primary diseases affecting the liver, kidneys, hematopoietic system, endocrine system, or other major organs; (3) history of lumbar spine surgery; (4) pregnant or breastfeeding women. Control group participants were asymptomatic volunteers recruited from the clinic during the same period, meeting the following inclusion criteria: (1) no history of chronic low back pain or any spinal disorders; (2) aged between 18 and 40 years; (3) no prior history of lumbar spine surgery or major musculoskeletal injuries; (4) no serious primary diseases affecting major organs; (5) provided signed informed consent. A total of 62 subjects, comprising 30 with CNLBP and 32 individuals without LBP, aged between 20 and 38 years, completed the study, including all basic information and relevant questionnaires. All participants followed the entire experimental procedure, and none were excluded from the analysis.

### 2.2 Examiners

All informed consent processes, screenings, and imaging procedures in this study were conducted jointly by one spine surgeon and two physical therapists. All examiners received specialized training in musculoskeletal ultrasound and low back pain assessment, as well as detailed instruction on the specific imaging procedures used in this study. Additionally, the examiners demonstrated high consistency and reliability in measurements during a prior pilot study. To ensure data consistency, all imaging procedures were independently performed by the same primary examiner, while an assistant was responsible for freezing the images as directed by the primary examiner. The assistant did not participate in the actual measurement process to avoid influencing data collection.

### 2.3 Pain and dysfunction assessment

The extent of low back pain was assessed using the NRS, where higher scores indicate more severe pain. This scale is widely used in pain research and clinical practice and is known for its sensitivity, validity, and reliability (Williamson and Hoggart, 2005). Additionally, the ODI was used to evaluate dysfunction in daily activities caused by low back pain (Fairbank and Pynsent, 2000). The sexual life item was excluded from this study, and the sum of the remaining items was expressed as a percentage, with higher percentages indicating greater dysfunction.

### 2.4 Shear wave elastography imaging

**FIGURE 1:**
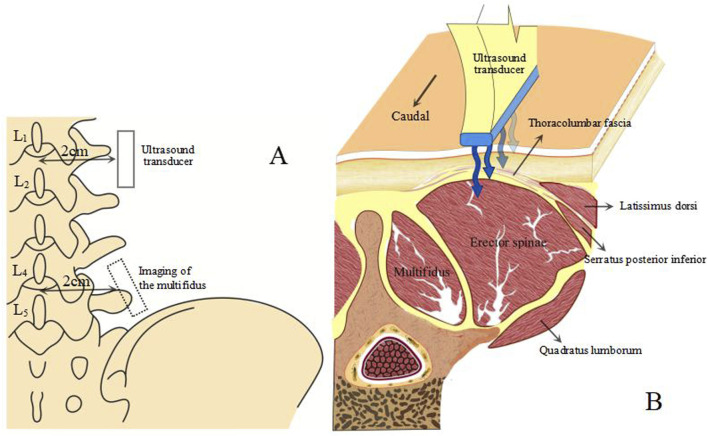
Ultrasound image acquisition method. **(A)** position of the ultrasound transducer relative to the spine. **(B)** anatomical cross-sectional image of the TLF, back and abdominal wall muscles.

Diagnostic color Doppler ultrasound (Mindray, Eagus R9, China) with an L11-3U high-frequency linear array probe was used for SWE imaging (Figure 1). Participants lay prone with their upper limbs flat by their sides, maintaining relaxation of their lower back muscles. A pillow was placed under their abdomen to minimize lumbar lordosis and restrict lumbar spine motion (Stokes et al., 2007). The Eagus R9 was set to SWE mode, with the preset elasticity range for tissue Young’s modulus visualization from 0 to 160 kPa, corresponding to a shear wave velocity range of 0–8.2 m/s. A 20 × 20 mm square inspection frame was set up at a depth of 3.5–8.0 cm from the skin surface, depending on the subject’s fat layer thickness. Images were frozen after the Motion Stability Index (M-STB Index) reached level 5. At the levels of the fourth and first lumbar vertebrae, points were marked bilaterally about 2 cm paramedian to the spinous processes, corresponding to the interspaces between the transverse processes of the L_4-5_ and L_1-2_ vertebrae. These positions were confirmed using ultrasound images. The probe was placed on the skin with a coupling agent to minimize the gap between the probe and the skin, ensuring no pressure was applied during contact (Cortez et al., 2016).

**FIGURE 2:**
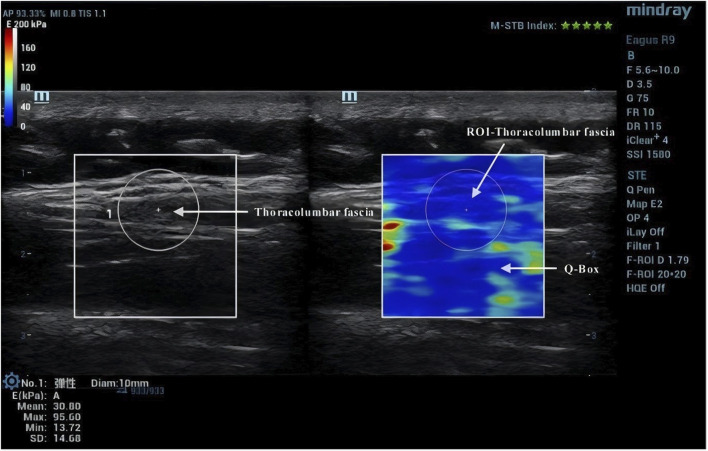
Region of interest for TLF shear wave imaging.

**FIGURE 3:**
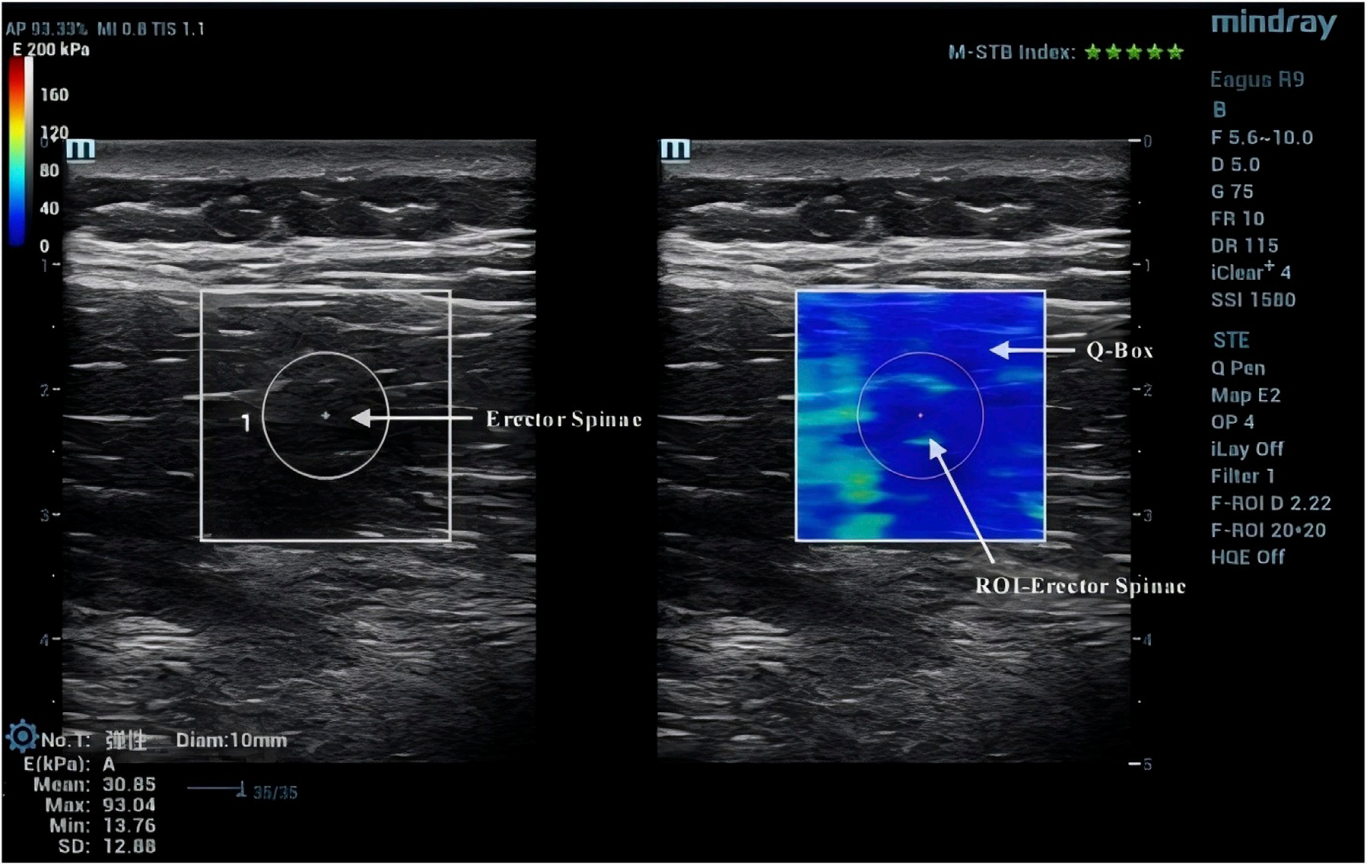
Region of interest for ES shear wave imaging.

**FIGURE 4:**
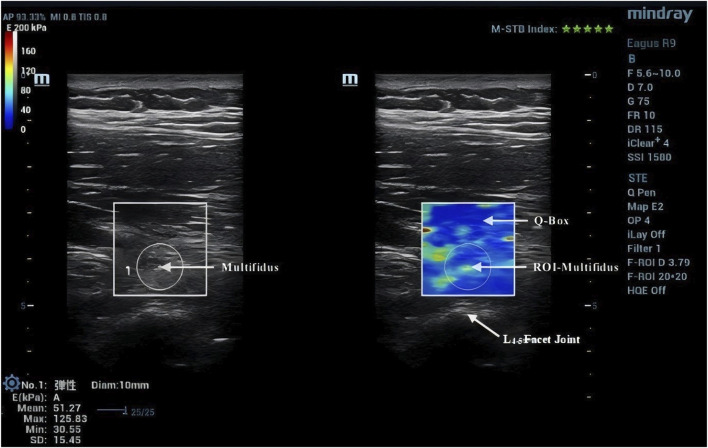
Region of interest for MF shear wave imaging.

Due to the anisotropic nature of muscle tissue, adjustments were made to the probe to ensure it was oriented parallel to the muscle fibers. Once the correct orientation was established, the outline of the probe was marked on the participant’s skin to maintain consistency in placement during measurements (Creze et al., 2017). TLF was clearly visible beneath the fat layer (Figure 2). For the erector spinae ES, located below the TLF, the course of its muscle fibers was easily identifiable (Figure 3). However, for the MF, which lies below the ES and above the articular processes of the vertebrae, the muscle fibers’ course was not clearly discernible. Instead, the MF was localized based on its anatomical position (Figure 4).

### 2.5 Image processing

After acquiring images using SWE, the Q-Box function was activated to define the region of interest (ROI), which was set as a circle with a diameter of 10 mm. The system then automatically calculated the average Young’s modulus value of the muscle tissue within the ROI. Q-Box excludes elastograms with artefacts caused by attenuation effects to avoid inaccurate elasticity measurements (MacDonald et al., 2016). The ROI’s size and position must clearly distinguish between the fascial plane, bony prominence, and any grey pixels. Bony prominences, being harder tissues than muscle and fascia, can significantly impact the outcome. Grey pixels represent areas where the Eagus R9 could not determine the shear wave propagation velocity, assigning these areas a value of zero. Once the Q-Box is accurately positioned, the average Young’s modulus value for each test can be recorded. The Young’s modulus (E, unit: kPa) within each ROI is automatically calculated by the SWE software using formula **E = 3ρV_s_^2^**, where **ρ** is the muscle tissue density (assumed to be 1,000 kg/m³) and V_s_ is the shear wave propagation velocity. Since muscle is highly anisotropic, its stiffness is usually calculated by dividing Young’s modulus by 3, resulting in the shear modulus **μ = ρV_s_^2^** (Gennisson et al., 2013).

### 2.6 Statistical analysis

The Shapiro-Wilk test was used to test whether the data were normally distributed. Differences between groups were tested using the independent samples *t*-test (normal distribution) or the Mann-Whitney U-test (non-normal distribution), and Cohen’s *d* (normal distribution) or η^2^ (non-normal distribution) were used as effect sizes, respectively. Thresholds for Cohen’s *d* were as follows: <0.20 (trivial), 0.21–0.50 (small), 0.51–0.80 (medium), >0.81 (large). η^2^ thresholds were as follows: 0.01–0.059, small; 0.06–0.14, medium; >0.14, large.

Pearson’s (normal distribution) or Spearman’s (non-normal distribution) correlations were used to analyze the relationship between the NRS pain score and ODI dysfunction index with the stiffness of the TLF, ES, and MF, while controlling for the covariates, age, height, weight, and BMI. Thresholds for correlation coefficients (r) were 0–0.1 (trivial), 0.1–0.3 (small), 0.3–0.5 (medium) and >0.5 (large) (Levin, 1988). All statistical analyses were completed using *IBM SPSS 27.0*, with the significance level set at 0.05.

## 3 Results

### 3.1 Fascia and muscle stiffness

T-tests revealed no significant differences between the two groups in terms of age, gender, height, weight, and body mass index (BMI). Detailed descriptive information on demographic variables and outcome measures is presented in Table 1. The results of intergroup comparisons of TLF, ES and MF shear modulus are shown in Table 2. Compared with the control group, the shear modulus values of the bilateral TLF of L_4-5_ were significantly higher in the CNLBP group (*p* = 0.014, 0.002, Cohen’s *d* = 0.64, 0.86). The results of bilateral intergroup comparisons showed that the ES shear modulus values of L_4-5_ bilaterally and L_1-2_ right side in the CNLBP group were higher than those of the control group and significantly different (*p* = 0.013, 0.027, 0.026, Cohen’s *d* = 0.66, 0.58, 0.59). Comparison of MF showed that shear modulus values were significantly higher in the CNLBP group for the L_4-5_ bilaterally and the left side of L_1-2_ than in the healthy control group (*p* = 0.009, 0.002, 0.020, Cohen’s *d* = 0.69, 0.85, η^2^ = 0.09).

**TABLE 1:** Characteristics of the participants.

Characteristic	CNLBP group		Healthy group		*p values*
	n = 30	Range	n = 32	Range	
Age (years)	27.40 ± 4.57	22–37	27.94 ± 4.94	20–38	0.659
Sex (male/female)	19/11	-	15/17	-	0.193
Height (m)	1.73 ± 0.66	1.62–1.83	1.70 ± 0.80	1.56–1.85	0.076
Weight (kg)	69.30 ± 11.64	52.21–89.26	65.17 ± 9.51	50.64–82.52	0.130
BMI (kg/m^2^)	22.96 ± 2.55	19.18–28.17	22.52 ± 2.26	18.70–27.55	0.480
Pain duration (months)	7.53 ± 3.22	3–15	-	-	-
NRS (1–10)	3.87 ± 1.31	2–7	-	-	-
ODI%	15.33 ± 6.02	6.67–33.33	-	-	-

NRS, numeric rating scale; ODI, oswestry disability index.

**TABLE 2:** Comparison of fascia and muscle stiffness between CNLBP and healthy groups.

Variables		CNLBP		Control		*P*	*d*	*η* ^*2*^
		Descriptive statistic	Range	Descriptive statistic	Range			
TLF L_4-5_	Left	12.29 ± 6.18	3.02–24.92	8.75 ± 4.73	2.54 ± 19.61	0.014*	0.64	-
	Right	12.55 ± 7.07	2.79–28.09	7.73 ± 3.81	2.10–16.58	0.002*	0.86	
TLF L_1-2_	Left^a^	11.25	7.58	8.97	6.71	0.098	-	0.04
	Right	12.47 ± 7.14	3.75–29.56	9.43 ± 4.89	2.69–24.13	0.054	0.50	-
ES L_4-5_	Left	13.11 ± 5.23	6.15–24.35	10.16 ± 3.55	3.87–18.94	0.013*	0.66	-
	Right	13.06 ± 4.84	5.50–23.52	10.65 ± 3.36	4.04–17.92	0.027*	0.58	-
ES L_1-2_	Left	12.61 ± 5.12	5.06–23.40	10.54 ± 3.77	3.86–20.69	0.077	0.46	-
	Right	13.11 ± 4.78	6.22–22.69	10.65 ± 3.51	4.38–18.20	0.026*	0.59	-
MF L_4-5_	Left	14.23 ± 5.09	2.84–29.01	11.29 ± 3.33	5.90–17.73	0.009*	0.69	-
	Right	13.97 ± 4.82	3.53–24.43	10.64 ± 2.83	5.10–18.57	0.002*	0.85	-
MF L_1-2_	Left^a^	12.97	3.20	11.28	4.28	0.020*	-	0.09
	Right	12.91 ± 3.32	3.46–20.02	11.43 ± 3.07	5.52–18.80	0.072	0.47	-

Unless otherwise stated, values are expressed as mean ± SD.

^a^ Data are presented as median, interquartile differences. *Statistically significant difference.

### 3.2 Correlation analysis

The results of the correlation of NRS scores with TLF, ES, and MF shear moduli are shown in Table 3. The shear moduli of TLF on the L_4-5_ bilaterally and on the right side of L_1-2_ showed moderate to strong correlation with NRS scores. The shear moduli of ES on the L_4-5_ bilaterally showed a moderate correlation with the NRS scores. Shear modulus of MF showed moderate correlation with NRS scores on the L_4-5_ bilaterally and on the left side of L_1-2_.

**TABLE 3:** Correlation between stiffness indicators and pain in the CNLBP group.

Variables		Pain (NRS)	
		*r*	*p*
TLF L_4-5_	Left	0.57	0.001*
	Right	0.65	< 0.001*
TLF L_1-2_	Left^a^	0.33	0.072
	Right	0.47	0.009*
ES L_4-5_	Left	0.42	0.022*
	Right	0.48	0.007*
ES L_1-2_	Left	0.33	0.075
	Right	0.31	0.098
MF L_4-5_	Left	0.50	0.005*
	Right	0.42	0.023*
MF L_1-2_	Left^a^	0.50	0.005*
	Right	0.36	0.051

^a^ Spearman correlation test was used. *Statistically significant difference.

The results of the correlation of ODI with the shear modulus of TLF, ES and MF are shown in Table 4. The shear modulus of TLF on the L_4-5_ bilaterally showed a strong correlation with ODI, and a moderate correlation was found on the right side of L_1-2_. Shear modulus of ES showed moderate correlation with ODI on the right side of L_1-2_. A moderate correlation was found between shear modulus and ODI for both L_4-5_ bilateral and L_1-2_ right side MF.

**TABLE 4:** Correlation between stiffness index and dysfunction in CNLBP group.

Variables		Dysfunction (ODI)	
		r	*p*
TLF L_4-5_	Left	0.60	< 0.001*
	Right	0.58	< 0.001*
TLF L_1-2_	Left^a^	0.35	0.056
	Right	0.43	0.017*
ES L_4-5_	Left	0.36	0.054
	Right	0.31	0.093
ES L_1-2_	Left	0.36	0.053
	Right	0.38	0.037*
MF L_4-5_	Left	0.46	0.011*
	Right	0.45	0.012*
MF L_1-2_	Left^a^	0.31	0.097
	Right	0.44	0.015*

^a^ Spearman correlation test was used. *Statistically significant difference.

## 4 Discussion

This study assessed differences in bilateral TLF, ES, and MF stiffness between subjects with CNLBP and healthy controls. Additionally, it examined the correlation between fascial and muscle stiffness and pain and dysfunction within the CNLBP group. Our findings indicate that TLF, ES, and MF stiffness were significantly higher in patients with CNLBP compared to those without low back pain. There was a positive correlation between increased stiffness and pain levels, which supports previous hypotheses. A significant positive correlation was found between increased stiffness and both pain severity and functional impairment index in certain measurement areas. Moreover, we observed a higher correlation between muscle and fascial stiffness and pain levels in the L_4-5_ region compared to L_1-2_. This may be attributed to the anatomical and functional characteristics of the lumbar spine. The load on the lumbar spine increases progressively from top to bottom. The L_4-5_ segment, located in the lower lumbar region, serves as a key junction for supporting body weight and transmitting forces. This increased mechanical load makes it more susceptible to degenerative changes in the joints and surrounding soft tissues. Additionally, the L_4-5_ segment is a critical point for spinal mobility, with significantly greater range of motion than the upper lumbar segments, facilitating complex movements such as flexion, extension, lateral bending, and rotation (Sabnis et al., 2018). Due to the higher frequency of movement, the L_4-5_ segment is more prone to chronic damage to the muscles, ligaments, and joints from repeated mechanical stress (Basques et al., 2017). Moreover, studies have shown that the L_4-5_ segment is a high-risk area for degenerative lumbar diseases, such as disc herniation, spinal stenosis, and osteoarthritis (Kurowski and Kubo, 1986; Liu et al., 2017). These long-term degenerative changes further increase the risk of injury to the surrounding fascia and muscles.

In this study, we found that the stiffness of the TLF was significantly increased in CNLBP patients, and there was a strong positive correlation between increased stiffness and pain and functional impairment, particularly in the L_4-5_ region. Several factors may contribute to these changes. First, prolonged mechanical stress and load are key factors. Chronic low back pain patients often experience sustained mechanical load on the fascia due to poor posture or repetitive stress, leading to fascia hypertrophy and fibrosis (Ramsook and Malanga, 2012). Second, fibrosis is a pathological change induced by chronic inflammation, where prolonged inflammation promotes the proliferation of fibrous tissue, reducing fascia elasticity and increasing stiffness (Kondrup et al., 2022). The decreased ability of the fascia layers to glide may also be a contributing factor to increased fascia stiffness, as the loss of elasticity restricts normal kinematic function, thereby affecting lumbar flexibility. Moreover, the myofascial system is critical for cushioning and force transmission in the human body. Unlike the rigid bones that act as pressure-resistant structures, these tissues rely on their tensile properties to function properly. When tissues lose their normal physiological elasticity, embedded receptors may remain active even at rest. In this non-physiological state, any muscle contraction or stretch may be transmitted through myofascial connections to neighboring tissues, leading to excessive sensory input (Sinhorim et al., 2021; Ushida et al., 2022). Fascial tissues, particularly the TLF, are rich in sensory nerve endings and innervation and may contain high-threshold mechanoreceptors (Suarez-Rodriguez et al., 2022). Wilke et al. (Wilke et al., 2017) pointed out that the mechanoreceptors densely distributed in fascia respond to applied pressure, leading to reduced sympathetic tone and changes in local tissue viscosity when stimulated. Previous studies have shown that pain often spreads outward from the initially affected area (Mosabbir, 2022). As other muscles compensate for the dysfunction, this compensation can result in overload, further expanding the impact of pain and dysfunction and causing discomfort over a broader range.

Additionally, as a crucial component of the superficial stabilizing muscle group, the ES plays a vital role in maintaining the stability of the spine and pelvis (Daggfeldt et al., 2000; Howarth et al., 2013). Due to its superficial location and ease of measurement, surface electromyography (sEMG) and B-mode ultrasonography are the most commonly used methods for assessing ES function. sEMG provides valuable information on neuromuscular electrical activity; however, its accuracy can be easily influenced by external factors and it does not reflect the biomechanical properties of the tissue. Consistent findings from sEMG studies suggest that ES activation levels are typically high, with a tendency for delayed activation (Becker et al., 2018; Sakai et al., 2019). B-mode ultrasound assesses muscle contraction by comparing muscle thickness or cross-sectional area at rest and during contraction (Cheung et al., 2020). However, significant errors may arise when the muscle cannot be assumed to be ‘at rest,’ such as in cases of muscle spasm or postural tension. The MF, located at the deepest level of the spinal region, has the largest attachment area among the paravertebral muscles and is rich in sensory receptors, playing a key role in spinal stability (Hofste et al., 2020). Research indicates that MF contributes to two-thirds of spinal stiffness during movement (Ward et al., 2009). Mechanical overload, prolonged immobilization, and sports injuries can lead to degenerative changes in the MF, including atrophy and fatty infiltration, resulting in delayed pre-activation, impaired coordination, and reduced control (Goubert et al., 2016). Numerous studies have linked MF degeneration and functional abnormalities to various spinal disorders, including chronic low back pain and degenerative lumbar spinal stenosis (Goubert et al., 2017; Faur et al., 2019). However, the relationship between morphological changes in the lumbar MF and lumbar spine function in CNLBP patients remains unclear. A cross-sectional study found a significant negative correlation between the severity of MF fatty infiltration and lumbar flexion mobility (Hildebrandt et al., 2017). In contrast, Rezazadeh et al. (Rezazadeh et al., 2019) reported no association between changes in MF cross-sectional area and dysfunction indices in CNLBP patients. Given these conflicting findings, a comprehensive assessment of lumbar function and pain in CNLBP patients is essential. Exploring MF changes from a biomechanical perspective may offer deeper insights into its role in CNLBP and inform more effective treatment strategies.

Based on SWE imaging, this study found that the stiffness of the ES and MF muscles was significantly higher in certain regions of CNLBP patients compared to those without low back pain, and this was correlated with pain and functional impairment. Chronic low back pain often triggers a “protective muscle contraction” mechanism, where the ES and MF muscles remain in a state of continuous contraction to limit excessive spinal motion and prevent further injury (Larsen et al., 2018). Moreover, reflexive control of spinal movement depends on sensory-motor mechanisms regulated by mechanoreceptor input. These afferent nerves are present in various spinal tissues, including paraspinal muscles, interspinal muscles, interspinous ligaments, thoracolumbar fascia, intervertebral discs, and facet joint capsules (Ebenbichler et al., 2001; Holm et al., 2002). The afferent nerves in the discs and joint capsules have high mechanical thresholds and are only activated under severe loading conditions, while those in muscles, ligaments, and fascia can be activated at lower thresholds and have proprioceptive functions (Yamashita et al., 1993; Sekine et al., 2001). Zuriaga et al. (Sanchez-Zuriaga et al., 2010) found that prolonged spinal flexion impairs sensory-motor control mechanisms and reduces the protective function of back muscles over the spine. This effect is attributed to time-dependent “creep” in soft tissues rather than muscle fatigue, which aligns with the findings of (Shin et al., 2009). Static posture, mechanical overload, and sports injuries can lead to delayed pre-activation of paraspinal muscles, altering lumbar load distribution and subsequently impairing coordination and control (Haddas et al., 2016). Nociceptors transmit neural impulses via Aδ and C fibers to the dorsal horn of the spinal cord, projecting to higher centers and triggering reflexive activity that produces pain. Concurrently, this reflex activity increases the excitability of α and γ motor neurons in the corresponding segments, enhancing the stretch reflex, which leads to muscle spasms, increased tension, and greater stiffness (Freddolini et al., 2014; Sessler et al., 2021; Creighton et al., 2023). Prolonged noxious stimulation of the lumbar muscles in CNLBP patients reduces the firing frequency of motor neurons, affecting the recruitment rate of sarcomeres, reducing the number of active thick filaments, and decreasing lumbar muscle contractility, which reflexively inhibits normal muscle activity (Russo et al., 2018; Noonan and Brown, 2021). These changes significantly reduce lumbar muscle involvement, disrupt spinal stability and vertebral balance, and create a vicious cycle that contributes to the development of low back pain.

The use of SWE in musculoskeletal applications, particularly for assessing muscular tissues, has been rapidly increasing since 2010 when initial studies demonstrated plausible changes in muscle stiffness using this technology (Shinohara et al., 2010). A previous study employed strain ultrasonography to evaluate muscle stiffness in the low back muscles, focusing solely on the MF. This study found no significant difference in lumbar MF stiffness between patients with low back pain and healthy subjects in the prone position (Chan et al., 2012). However, quantitative measurement of muscle stiffness using strain-based imaging methods poses challenges. The accuracy of these methods depends heavily on the compression control exerted by the assessor on the probe, and they do not provide absolute values. In contrast, SWE offers quantitative measurements that are less dependent on the assessment technique, resulting in more accurate and reliable results. SWE is increasingly recognized as the best method for estimating individual muscle strength and can quantify localized changes in muscle damage, such as myofascial trigger points (Hug et al., 2015). The validity and reliability of SWE for assessing the TLF, ES, and MF are well-established, but most studies have focused on asymptomatic subjects (Koppenhaver et al., 2018; Chen et al., 2020). Only two similar studies on low back pain have been found, and their outcomes are inconsistent. Masaki et al. reported significantly higher MF stiffness in patients with LBP compared to individuals without LBP, with no significant difference in ES stiffness. However, this study included only nine patients, which could impact the reliability of the results (Masaki et al., 2017). Another study found that both ES and MF stiffness were significantly higher in LBP patients than in asymptomatic controls, with a positive correlation between stiffness, pain, and dysfunction (Koppenhaver et al., 2020). Additionally, neither study differentiated between NLBP and specific low back pain, which includes lumbar spine disorders and may present differently. In our study, we specifically identified patients with CNLBP and aimed to assess the stiffness of muscles and fascia in multiple lower back areas. To our knowledge, this is the first study to compare TLF stiffness between CNLBP patients and asymptomatic subjects and to correlate it with clinical symptoms.

Quantifying the elasticity index of muscles and fascia in patients with CNLBP and analyzing its correlation with clinical symptoms from a biomechanical perspective not only helps clarify the predisposing factors of tissue pain and injury but also aids in understanding the integrity of the body’s tissue structure. This approach provides valuable insights for diagnosing low back pain and assessing the efficacy of treatment interventions. The stiffness of lumbar muscles and fascia may serve as a crucial diagnostic or prognostic factor for CNLBP, offering a potential metric for evaluating the condition’s progression and response to treatment.

## 5 Limitation

This study presents several limitations that warrant consideration. First, the cause of the increased lumbar fascial or muscle stiffness observed in patients with CNLBP remains uncertain whether it stems from overuse, muscle spasm, or another factor. In future studies, SWE combined with electromyography could be used to evaluate the stiffness characteristics of lumbar fascia and muscles under different postures or contraction states. Second, the mechanisms underlying pain generation and adaptation are complex. Given that this study is cross-sectional, the correlational conclusions drawn from it should be interpreted with caution. Future research could benefit from longitudinal studies that assess changes in fascial and muscle stiffness before and after specific interventions using SWE. Thirdly, the study’s participants were all recruited from the same geographical area and primarily consisted of young to middle-aged adults between 20 and 35 years. Therefore, caution should be exercised when extrapolating these findings to other demographic groups, as the results may not be generalizable to older adults or populations from different regions.

## 6 Conclusion

In patients with CNLBP, the stiffness of the TLF, ES, and MF is generally higher than in individuals without low back pain. However, this increased stiffness is not consistently observed across all detection areas. Additionally, higher stiffness may be associated with pain and dysfunction, with this relationship being more pronounced in the TLF.

## Data Availability

The raw data supporting the conclusions of this article will be made available by the authors, without undue reservation.

## Abbreviations

TLF, Thoracolumbar fascia; ES, Erector spinae; MF, Multifidus; CNLBP, Chronic non-specific low back pain; SWE, Shear wave elastography; NRS, Numeric rating scale; ODI, Oswestry disability index; NLBP, Non-specific low back pain; ROI, Region of interest.
